# 528 Evaluation of phosphate replacement practices in burn patients

**DOI:** 10.1093/jbcr/irac012.158

**Published:** 2022-03-23

**Authors:** Giae Surine S Derisse, Elizabeth Panter, Angela M Helder, Nicole D Kiehle

**Affiliations:** University of Maryland Saint Joseph Medical Center, Baltimore, Maryland; Johns Hopkins Bayview Medical Center, Baltimore, Maryland; Johns Hopkins Bayview Medical Center, Baltimore, Maryland; University of Maryland Baltimore Washington Medical Center, Glen Burnie, Maryland

## Abstract

**Introduction:**

Burn injury causes acute shifts in phosphorus leading to hypophosphatemia and negative sequelae, such as motor neuropathy, muscle weakness, cardiac failure, and respiratory failure. These patients require frequent phosphorus level monitoring and repletion beyond the needs of a general critical care patient. Data suggests patients with normal phosphorus levels have lower incidence of ventilator wean failure and positive clinical outcomes. There is limited data evaluating phosphate replacement practices in burn patients for their intensive care unit (ICU) length of stay while also evaluating those who concomitantly receive continuous renal replacement therapy (CRRT) as it is the primary mode of renal replacement therapy in this population and further depletes phosphorus levels.

**Methods:**

This was a single-center, retrospective, observational study of patients with a burn injury admitted and discharged from a burn intensive care unit (BICU) from January 1, 2016 to June 30, 2020 who received phosphate. Patients less than 18 years of age and those admitted to the BICU for non-burn injuries were excluded. Burn injury type, number of phosphorus doses per day, and phosphorus levels were collected. Normal phosphorus was defined as 2.5-4.9 mg/dL and hypophosphatemia as < 2.5mg/dL. Patient data was evaluated in 24-hour time intervals as defined as midnight to midnight. Phosphorus lab values were included in data analysis if there was corresponding phosphate administration in that 24-hour interval. The primary objective was to assess the temporal dose-response to phosphate replacement in burn patients.

**Results:**

There were 291 patients who met criteria, 116 were selected in chronological order by admission date and were included in data analysis. The mean age was 51.51 years and the mean total body surface area burned was 21.48%. Flame burn accounted for 83.6 % (n=97) of patients and 37.06% (n=43) of patients had concomitant inhalation injury. The mean amount of phosphate given to a patient per day was 28.38 mmol and patients on CRRT received a mean amount of phosphate of 33.34 mmol per day. In response to phosphate administration, the mean change in phosphorus was 0.334 ± 1.08 mg/dL. In patients on CRRT, the mean change in phosphorus was 1.4 ± 1.89 mg/dL. Patients experienced hypophosphatemia 69.63% of the days that they received phosphate repletion and patients on CRRT had hypophosphatemia 87.39% of the days they received phosphate repletion.

**Conclusions:**

Hypophosphatemia is common in the burn injury population and current phosphate replacement practices are insufficient to replete phosphorus in burn injury patients.

